# Tacrolimus Protects Podocytes from Apoptosis via Downregulation of TRPC6 in Diabetic Nephropathy

**DOI:** 10.1155/2021/8832114

**Published:** 2021-05-20

**Authors:** Ruixia Ma, Ying Wang, Yan Xu, Rui Wang, Xianghua Wang, Ning Yu, Minghui Li, Yan Zhou

**Affiliations:** ^1^Department of Nephrology, Affiliated Hospital of Qingdao University, Qingdao, Shandong, China; ^2^Department of Intensive Care Unit, Affiliated Hospital of Qingdao University, Qingdao, Shandong, China; ^3^Department of Ultrasound, Affiliated Hospital of Qingdao University, Qingdao, Shandong, China; ^4^Department of Emergency, Affiliated Hospital of Qingdao University, Qingdao, Shandong, China

## Abstract

Podocyte injury plays an important role in diabetic nephropathy (DN), and apoptosis is one of its mechanisms. The transient receptor potential channel 6 (TRPC6) is expressed in podocytes and mediates podocyte injury induced by high glucose levels. Tacrolimus is a novel immunosuppressive agent that is reported to play an important role in podocyte protection. The purpose of this study was to investigate the potential mechanism of podocyte protection by tacrolimus in a type 2 diabetic mellitus (T2DM) rat model and in immortalized mouse podocytes (MPC5). Transmission electron microcopy was used to evaluate renal injury morphology. After treatment with FK506, we measured 24-hour urinary albumin-to-creatinine ratios and creatinine clearance rates as well as major biochemical parameters such as glucose, insulin, serum creatinine, urea nitrogen, total cholesterol, triglycerides, alanine transaminase, and aspartate aminotransferase. Nephrin and TRPC6 protein expression and podocyte apoptotic rates *in vivo* and *in vitro* were measured using immunohistochemical staining, TUNEL assays, and flow cytometry, respectively. Western blot was used to measure expression of cleaved-caspase-3 and bax/bcl-2. Exposed to high glucose (HG), DM rats exhibited disrupted biochemical conditions and impaired podocyte structure. Decreased expression of nephrin and increased expression of TRPC6, cleaved-caspase-3, and bax/bcl-2 ratios were found in podocytes, along with higher apoptotic percentage, while tacrolimus intervention counteracted the effect of HG on podocytes. Our results suggest that tacrolimus protects podocytes during the progression of type 2 diabetic nephropathy, possibly ameliorating podocyte apoptosis by downregulating the expression of TRPC6.

## 1. Introduction

Diabetic nephropathy (DN) is one of the most common and serious microvascular complications of diabetic mellitus (DM); it is a leading cause of end-stage renal disease (ESRD) [1, 2]. Approximately 30%–40% of patients with DM develop renal disease [3, 4]. Currently, DN is attracting increased attention on the part of researchers because of the increasing number of new diagnoses as well as the difficulty of treatment, both of which impose socio-economic stress on healthcare systems worldwide. Nevertheless, little is known about pathogenesis of DN, which may be related to genetic factors, abnormal glucose and lipid metabolism, oxidative stress, immune response, podocyte injury, and other factors. Damage to podocytes is generally acknowledged as an important characteristic of DN that leads to proteinuria.

Podocytes are terminally differentiated visceral cells of glomeruli, a major part of glomerular filtration barrier (GFB), possessing limited regeneration probability [5]. Stimulation of high sugar, high fat, and various cytokines drives podocyte damage. Evidence shows a close relationship among podocyte injury, proteinuria, and progressive glomerulosclerosis, suggesting that podocyte injury plays a key role in DN [6]. Podocyte injury includes changes in either the morphology or the number of podocytes. The podocyte is composed of several parts: a cell body, primary process, and secondary and tertiary foot processes (FPs). Neighboring FPs are connected by a specialized cell junction called slit diaphragms (SDs), where nephrin, podocin, and transient receptor potential channels (TRPC) coexist [7]. Any abnormalities of cytoskeleton or SD can cause FP fusion, leading to GFB function loss and proteinuria. Susztak et al. [8] demonstrated that cell death contributed to podocyte loss in DN. Nevertheless, apoptosis has been described as the major mechanism by which podocytes die in diabetes and other progressive glomerular diseases [9–12]. Podocytes exposed to high glucose may have increased levels of reactive oxygen species (ROS), resulting in apoptosis by activation of MAPK and the caspase-3 family.

Transient receptor potential cation channel 6 (TRPC6) is an essential component of SDs, located in the podocyte membrane. A large body of data has indicated TRPC6 as a promising factor of podocyte depletion in various diseases [13]. First, gain-of-function mutations of TRPC6 were associated with the onset of familial forms of focal segmental glomerulosclerosis; furthermore, overexpression or overactivity of wild-type TRPC6 was sufficient to cause other kidney diseases [14–16]. Nevertheless, both increased TRPC6 channel expression and activity drive high levels of Ca^2+^ influx, eventually leading to podocyte loss. Recent evidence suggests that TRPC6 has a prominent role in the progression of DN. There appears to be a relationship between TRPC6 and podocyte injury. Angiotensin II, reactive oxygen species, and other factors occurring in the setting of DN may stimulate dramatic increases in calcium influx through the TRPC6 channel, causing podocyte injury [17].

Calcineurin inhibitors (CNIs) such as tacrolimus (FK506) are novel potential immunosuppressive agents, widely used for antiallograft rejection after organ transplantation and treatment of various immune system diseases. CNIs decrease the production of cytokines by inhibiting the nuclear factor of activated T cells (NFAT). The evidence suggests that immune dysfunction plays an essential role in pathogenesis of many kinds of kidney diseases, including several types of nephrotic syndrome that are caused by dysregulation of T-cell function. CNIs may protect podocytes through inhibition of NFAT activation, which is responsible for proteinuria and glomerulosclerosis [18]. The Kidney Disease: Improving Global Outcomes (KDIGO) Clinical Practice Guideline for Glomerulonephritis of 2012 suggested that CNIs should be used in treatment of idiopathic membranous nephropathy (IMN) [19]. Both tacrolimus combined with glucocorticoids and tacrolimus monotherapy are effective treatments of IMN, with significant decreases in 24-hour urinary protein excretion, increases in serum albumin, and estimated glomerular filtration rates (eGFR) [20, 21]. Wakamatsu et al. showed that FK506 significantly ameliorates proteinuria in an antinephrin antibody-induced nephrotic rat model [22].

Whereas podocyte injury leads to increased urinary protein excretion and FK506 reduces podocyte damage, the mechanisms remain unclear: there may be improved podocyte-specific expression of proteins such as nephrin and podocin [23] or inhibition of podocyte epithelial-mesenchymal transition (EMT) [24]. Our previous studies demonstrated that FK506 protects DN podocytes by upregulating autophagy [25] and downregulating oxidative stress [26]. Nevertheless, there are very few studies regarding FK506 and podocyte apoptosis in DN. Therefore, in this study, we focused on the effect of FK506 on podocytes *in vivo* and *in vitro*. We aimed to determine whether FK506 protected podocyte, and if so, how it achieved this effect, and whether FK506 reduces podocyte apoptosis.

## 2. Materials and Methods

### 2.1. Animals

Eight-week-old male Wistar SPF rats (weight 250–280 g, provided by Qingdao Animal Experimental Center, certificate number SCXK (Lu) 20080010) were housed in individual cages in a temperature-controlled room (20 ± 1°C, relative humidity 45–65%) and had free access to food and water under a 12 h light/dark cycle. To establish the T2DM model, rats were fed a high-calorie diet (10% animal fat, 20% cane sugar, 2.5% cholesterol, 1% cholate, and 66.5% regular chow), as previously described [27–29]. After 8 weeks, the rats fed the high-calorie diet were intraperitoneally administered a low dose of streptozotocin (30 mg/kg, Sigma, St. Louis, MO, USA). We used the insulin sensitivity index (ISI) (ISI = 22.5/FBG (fasting blood glucose) × FINS (fasting serum insulin), HOMA method) to determine whether the T2DM model was established.

The rats fed with high-calorie diet and intraperitoneally administered streptozotocin mentioned above were then divided into the DM group (diabetes mellitus, *N* = 10) and the FK group (FK506, *N* = 10). We also established the NC group (normal control, *N* = 10). Gastric gavage of FK506 (0.5 mg/(kg·d), Astellas Ireland Co., Ltd.) for the FK506 group or equal volume of citrate buffer solution for the NC and DM groups was administered once a day for 8 weeks, and all rats were given a normal caloric diet. Then, the body weights (BWs) were recorded, and urine samples were collected. Subsequently, the rats were sacrificed to collect the blood samples and kidneys. The experimental protocols were approved by the Ethics Committee of Affiliated Hospital of Qingdao University (number QYFYWZLL 25660).

### 2.2. Cell Culture

Conditionally immortalized mouse podocytes (MPC5) were cultured as described previously [30]. Cells were cultured under growth-permissive conditions at 33°C with 5% CO_2_ in RPMI-1640 medium (Hyclone, USA) supplemented with 10% fetal bovine serum (Hyclone), 10 U/mL mouse recombinant interferon-gamma (IFN-*γ*; PeproTech USA), and 100 U/mL penicillin plus 100 mg/mL streptomycin (Sigma). To induce differentiation, podocytes were maintained in nonpermissive conditions at 37°C in the absence of IFN-*γ* for at least 2 weeks and then were used for experiments. After serum starvation for 24 hours, differentiated podocytes were divided into four groups: NG group (normal glucose, 5.5 mmol/L), HG group (high glucose, 30 mmol/L), HM group (NG + mannitol 25 mmol/L), and FK group (HG + FK506 5 *μ*g/mL). After 48 hours, expression levels of nephrin, cleaved-caspase-3, and bax/bcl-2 proteins were measured using immunohistochemical staining and Western blot analysis.

### 2.3. Measurement of Biochemical Parameters

Serum insulin levels were determined using an enzyme-linked immunosorbent assay (ELISA) kit (Aquatic Diagnostic Ltd & Glasgow, Scotland). Fasting blood glucose (FBG), serum creatinine (Scr), blood urea nitrogen (BUN), total cholesterol (TC), triglyceride (TG), aspartate transaminase (AST), and alanine transaminase (ALT) levels were determined using a 7020 biochemical analyzer (Hitachi Ltd., Tokyo, Japan) at the Department of Clinical Laboratory, Affiliated Hospital of Qingdao University. The counts of white blood cells in blood samples were measured using a blood cell analyzer (Prang Medical, China). All biochemical parameters were examined at the 8th week after administration of FK506.

### 2.4. Determination of Urinary Albumin and Creatinine Concentrations

To determine urine albumin (UAL) and creatinine (Ucr) concentrations, 24 h urine samples were collected before and 8 weeks after administration of FK506. UAL excretion was measured using an enzyme-linked immunosorbent assay (ELISA) kit (Aquatic Diagnostic Ltd & Glasgow, Scotland). sUcr levels were measured using an automatic biochemistry analyzer (Hitachi Ltd., Tokyo, Japan). Twenty-four-hour urinary albumin-to-creatinine (uACR) and creatinine clearance (Ccr) were then calculated (Ccr = UCr/Scr × *V*, *V*: mL/24 h, urine in 24 h).

### 2.5. Noninvasive Blood Pressure Measurement

Blood pressure was measured using the tail-cuff method (LE5002, DSL, USA) in resting, conscious condition in a climate-controlled room (23°C). Systolic blood pressure (SBP) was consecutively measured five times, and the average of five readings was recorded.

### 2.6. Light and Transmission Electron Microscopy

Body mass and mass of the left kidneys were weighed before and after sacrifice, respectively. The kidney hypertrophy index was calculated by the ratio of kidney mass (KM) to body mass (BM). Kidneys were harvested and fixed in 10% paraformaldehyde, dehydrated, and embedded in paraffin. Thin sections of tissues were created for hematoxylin-eosin (HE), periodic acid-Schiff base (PAS), and Masson's trichrome staining. Pathological changes were examined using a light microscope.

Renal specimens were fixed in 2.5% glutaraldehyde and then dehydrated. After staining using uranyl acetate-lead citrate, the morphological characteristics of renal cortex sections (50 nm), including the thickness of GBM, and the condition of podocytes were measured using transmission electron microscopy. Renal specimens were photographed using a JEM-1200 transmission electron microscope (Jeol Ltd., Tokyo, Japan). The foot process fusion rate (FPR) was calculated as FPR (%) = *ΣL*_FP_/*ΣL*_BM_, where *ΣL*_FP_ is the total length of the podocyte fusion foot process and *ΣL*_BM_ is the total length of the peripheral capillary basement membrane.

### 2.7. Immunohistochemical Staining of Nephrin and TRPC6

Deparaffinized sections were stained with primary antibodies, specifically, rabbit-anti-rat nephrin antibody (1 : 200). For TRPC6, slides were incubated with goat anti-TRPC6 antibodies at 1 : 500 overnight at 4°C and then with biotinylated secondary antibodies (1 : 1000). All antibodies were purchased from Santa Cruz Biotechnology, Inc. (Santa Cruz, CA, USA). The color was developed by incubating with diaminobenzidine (Santa Cruz Biotechnology, Inc.) and counterstaining with hematoxylin. Semiquantitative immunohistochemical analysis was scored as follows: 0, absent or weak staining; 1, staining area < 25%; 2, staining area between 25 and 50%; 3, staining area between 50 and 75%; and 4, staining area > 75%. Ten glomeruli were examined randomly per slide to calculate the average integral value.

### 2.8. Western Blot

Cells and the homogenized renal cortex tissues were lysed in cold cell lysis buffer with protease and phosphatase inhibitors. The proteins were separated on 10% SDS-PAGE gels (10% separation gel, 6% concentrated gel) and subsequently electroblotted onto polyvinylidene fluoride (PVDF) membranes. Nonspecific antibody binding was blocked with preincubation in TBS containing 5% skim milk for 1 h at room temperature; then, membranes were incubated with primary antibodies against cleaved-caspase-3, bax, bcl-2, glyceraldehyde-3-phosphate dehydrogenase (GAPDH) (1 : 1000, all from Abcam), and TRPC6 (1 : 500-1 : 4000, rabbit anti-rat, Santa Cruz Biotechnology, Inc.) overnight at 4°C and then incubated with horseradish peroxidase- (HRP-) conjugated secondary antibody (1 : 10000, Abcam, Inc.). The blots were exposed using an enhanced chemiluminescence (ECL) system (Beijing Zhongshan Golden Bridge Biotechnology Co., Ltd., China).

### 2.9. Measurement of Apoptosis

A terminal deoxynucleotidyl transferase-mediated dUTP nick-end labeling (TUNEL) assay with an In Situ Cell Death Detection Kit (Roche, Germany) was used to determine apoptosis within glomeruli following the manufacturer's protocol. Briefly, kidney tissues were fixed in 4% paraformaldehyde, incubated with TUNEL reaction mixture, and then followed with antifluorescein-POD conjugate. The degree of apoptosis was estimated using a scale based on the mean number of TUNEL-positive cells per 100 glomerular sections.

Apoptosis *in vitro* was quantified using flow cytometry (FCM) after cell staining with an Annexin V-FITC Apoptosis Detection Kit I (BD PharMingen). Briefly, cells were washed twice with cold PBS and resuspended in binding buffer at 1 × 10^6^ cells/mL. Then, 5 *μ*L of FITC Annexin V and propidium iodide (PI) was added to 100 *μ*L cell suspensions for 15 min in the dark. Following the incubation, the mixture was diluted with 400 *μ*L binding buffer and analyzed by a BD Accuri C6 flow cytometer (BD Biosciences).

### 2.10. Statistical Analysis

Data were expressed as means ± SEM. Statistically significant differences were assessed using analysis of variance (one-way ANOVA). The LSD *t*-test was used for comparisons between every two groups. All statistical analyses were performed using a commercially available statistical package (SPSS 17.0). *P* values <0.05 indicated statistical significance.

## 3. Results

### 3.1. Biochemical Parameters

Levels of SBP, CCr, FBG, and ISI were significantly higher in the DM and FK groups than in the NC group; however, there were no significant differences between DM and FK groups (Table 1). The BWs and the KM/BM in the DM rats were significantly higher than those in NC rats. After using FK506, KM/BM of diabetic rats were statistically lower than those of the DM group. Furthermore, levels of lipid metabolic parameters such as TG and TC levels were significantly higher in the DM group than in the NC group; however, there were no significant differences between the untreated diabetic group and the FK506-treated group. There were no significant differences in levels of AST, ALT, and WBC counts following FK506 treatment, suggesting that the dose of FK506 had no influence on hepatic or bone marrow function.

### 3.2. 24-Hour Urinary Albumin-to-Creatinine Ratio (uACR)

**Figure 1:**
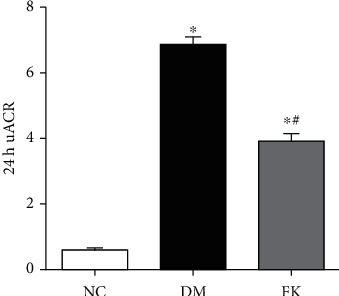
FK506 significantly ameliorated 24 h uACR of DM rats. ^∗^*P* < 0.05 compared to the NC group, ^∗^#*P* < 0.05 compared to the DM group.

The DM group had significantly greater 24 h uACR than did the NC group (6.83 ± 0.67 vs. 0.60 ± 0.25, *P* < 0.05), and this difference was suppressed by 8-week FK506 treatment (3.89 ± 0.69 vs. 6.83 ± 0.67, *P* < 0.05) (Figure 1).

### 3.3. Morphological Variation of Podocytes

**Figure 2:**
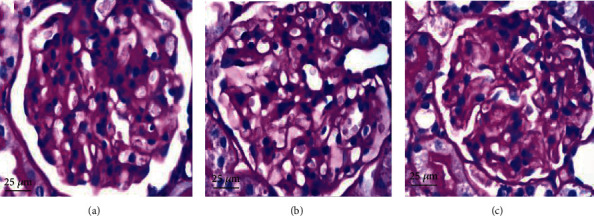
Histopathology of rat renal tissues. Periodic acid-Schiff base (PAS) staining of renal tissues from the (a) NC group, (b) DM group, and (c) FK group. Magnification ×400.

**Figure 3:**
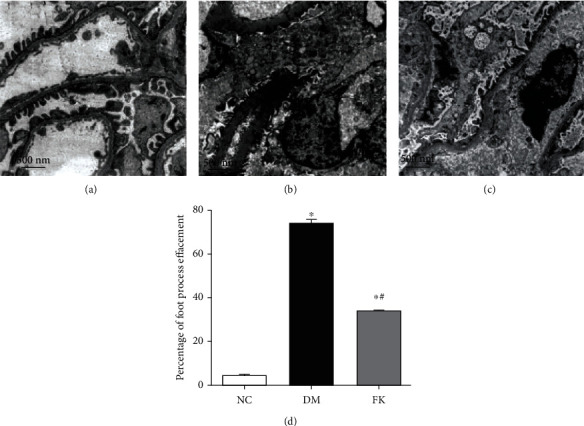
Effects of FK506 on ultrastructural variations of podocyte in DN rats. Three groups were included in this morphological observation, (a) NC group, (b) DM group, and (c) FK group, and the FK506 treatment improved the impaired ultrastructure of podocytes and foot process fusion rate (d) in DM rats. ^∗^*P* < 0.05 compared to the NC group, ^∗^#*P* < 0.05 compared to the DM group.

To determine morphology changes of podocytes, we used transmission electron microscopy. We found that foot processes of neighboring podocytes were interdigitated. There was no foot process fusion, and the thickness of the glomerular basement membrane was uniform in the NC group (Figures 2(a) and 3(a)). For the DM group, the glomerular basement membrane (GBM) was diffusely thickened, and foot process fusion rate was significantly greater (73.54 ± 3.43% vs. 4.26 ± 1.19%, *P* < 0.05, Figures 2(b), 3(b), and 3(d)). However, application of FK506 reduced the foot process rate (33.28 ± 1.91% vs. 73.54 ± 3.43%, *P* < 0.05) and the rate of thickened GBM were also ameliorated (Figures 2(c), 3(c), and 3(d)).

### 3.4. The Distribution and Expression of Nephrin and TRPC6

**Figure 4:**
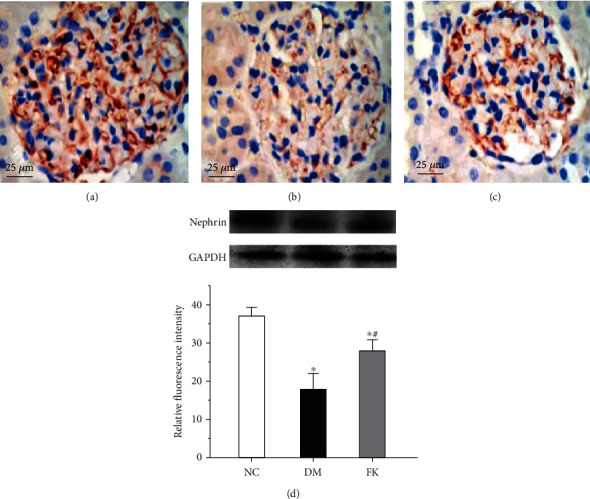
Podocyte-specific nephrin expressions detected by immunohistochemistry. Nephrin distribution patterns were observed in the (a) NC group, (b) DM group, and (c) FK group. (d) Western blot and quantification of nephrin integral values in each group. ^∗^*P* < 0.05 compared to the NC group, ^∗^#*P* < 0.05 compared to the DM group.

**Figure 5:**
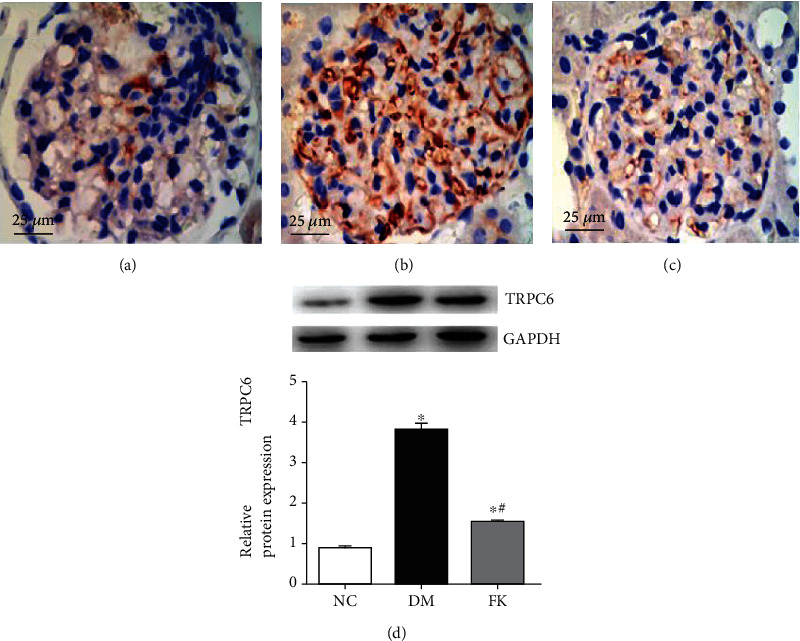
The distribution and expression of TRPC6 observed in the (a) NC group, (b) DM group, and (c) FK group. TRPC6 showed an increased staining intensity in DM rats while FK506 ameliorated this change. ^∗^*P* < 0.05 compared to the NC group, ^∗^#*P* < 0.05 compared to the DM group.

Immunohistochemistry in the kidney tissues revealed a uniformity and linear distribution of nephrin and less TRPC6 expression in the NC group, which was localized in glomeruli (Figures 4(a) and 5(a)). In the DM group, there was a significant decrease in nephrin expression levels, showing nonuniformity and nonlinear distribution (*P* < 0.05, Figure 4(b)) and significantly increased expression of TRPC6 (*P* < 0.05, Figure 5(b)). After 8-week treatment, the suppressed nephrin expression was significantly improved by FK506 treatment (*P* < 0.05, Figures 4(c) and 4(d)), as was the case for the increased TRPC6 expression (*P* < 0.05, Figures 5(c) and 5(d)).

### 3.5. Podocyte Apoptosis *In Vivo*

**Figure 6:**
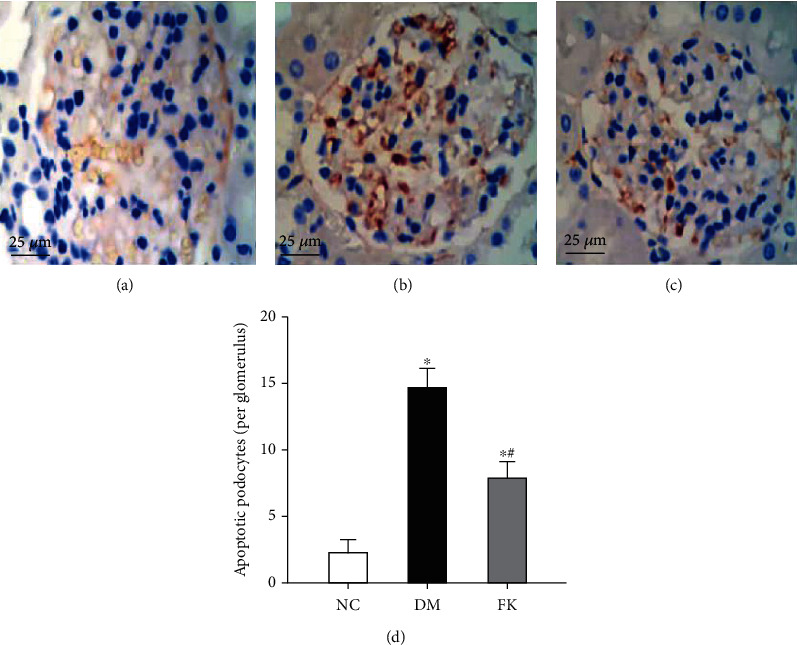
Podocyte apoptosis of rat renal tissues observed by TUNEL assay. (a) NC group, (b) DM group, and (c) FK group and (d) the counting of apoptotic podocytes per glomerulus in three groups. ^∗^*P* < 0.05 compared to the NC group, ^∗^#*P* < 0.05 compared to the DM group.

TUNEL assay was performed to identify podocyte apoptosis *in vivo.* The counts of apoptotic podocytes were significantly greater in the DM group than in the NC group (14.70 ± 1.20 vs. 2.20 ± 0.80 per glomerulus, *P* < 0.05, Figures 6(a) and 6(b)), while FK506 significantly ameliorated podocyte apoptosis (7.90 ± 1.10 vs. 14.70 ± 1.20 per glomerulus, *P* < 0.05, Figures 6(c) and 6(d)).

### 3.6. Effect of FK506 on Mouse Podocyte Apoptosis

**Figure 7:**
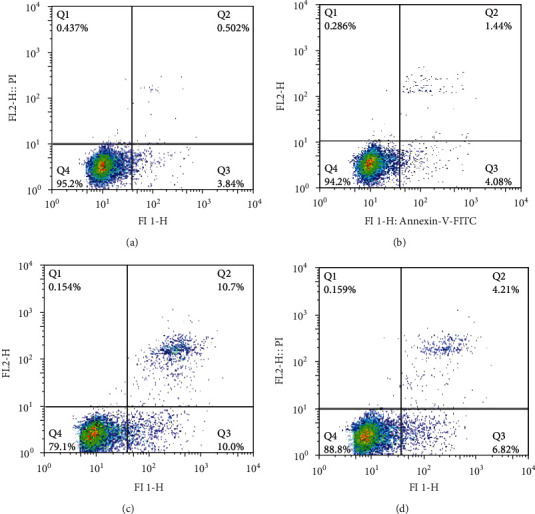
Effect of FK506 on podocyte apoptosis. (a) Cellular apoptosis under normal conditions. (b) Podocyte apoptosis of hypertonic group (HM). (c) Cellular apoptosis in the high glucose-treated group (HG), and (d) after using FK506, apoptotic podocyte counting was significantly decreased in the FK506 group.

To determine whether FK506 affected podocyte apoptosis, flow cytometry apoptosis assays were performed. We found that podocytes exhibited a significantly higher apoptotic percentage in high glucose-treated podocytes than that in normal conditions (Figures 7(a) and 7(c)). FK506 treatment significantly diminished podocyte apoptosis (Figure 7(d)).

### 3.7. Western Blot Analysis of Apoptotic Proteins

**Figure 8:**
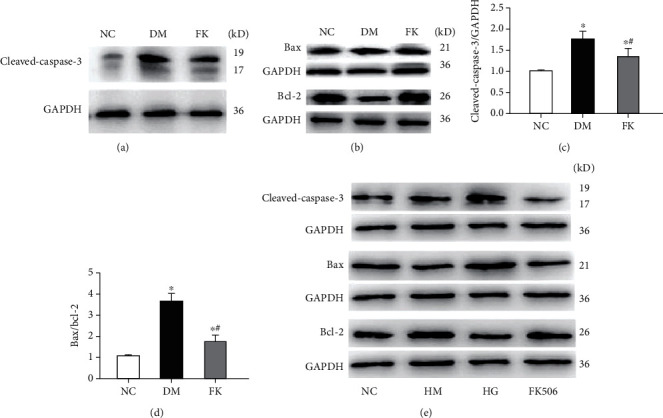
Western blot analysis of cleaved-caspase-3 and bax/bcl-2. Expressions of all above apoptotic proteins in the DM group were significantly increased compared with the NC group, and FK506 suppressed the apoptosis (a–d). Similar results were found in mouse podocytes (e). GAPDH: glyceraldehyde-3-phosphate dehydrogenase. ^∗^*P* < 0.05 compared to the NC group, ^∗^#*P* < 0.05 compared to the DM group.

Expression of cleaved-caspase-3 and the ratio of Bax to Bcl-2 represent responses to cell death signals and are considered indicators of the activation of apoptosis. Diabetic animals showed significantly greater level of kidney expressions of cleaved-caspase-3 and bax/bcl-2 ratios than did control rats (Figures 8(a) and 8(b)). FK506 administration had an antiapoptotic effect, as it prevented diabetes-induced increases in cleaved-caspase-3 levels and bax/bcl-2 ratios (*P* < 0.05, Figures 8(c) and 8(d)). *In vitro*, high glucose-treated and FK506-treated mouse podocytes exhibited the same results, while no differences were found between the HM and the NC groups (Figure 8(e)).

## 4. Discussion

We report here our recent findings about the protective role of FK506 on the podocyte apoptosis in DN, which maybe related to downregulation of TRPC6. We have utilized the rat model of DN on the basis of high-calorie diet and intraperitoneally administered streptozotocin. As shown in Table 1 and Figures 1, 2 and 3, DM rats exhibited impaired biochemical parameters and podocyte morphology. Increased expression of TRPC6, cleaved-caspase-3, bax/bcl-2, and higher apoptosis rate was detected together with decreased expression of nephrin. All of these can be improved after treatment of low dose of FK506, and more importantly, without significant effect on WBC counting and ALT and AST (Table 1).

The increasing prevalence and incidence of DN place heavy global socio-economic burdens on healthcare systems worldwide, being one of the main indications for hemodialysis. For this reason, it has become urgent to elucidate the underlying mechanisms of the pathogenesis of DN and to identify novel targets for early diagnosis and therapy. Podocytes are vital components of GFB, particularly because they cannot regenerate after suffering injury. Their apoptosis and structural damage result in the destruction of GFB with consequent proteinuria, both of which are decisive for prognosis of DN.

The mechanisms underlying podocyte injury in DN remain incompletely understood. Researchers have proposed several mechanisms, including podocyte hypertrophy, epithelial-mesenchymal transition (EMT), podocyte detachment, and apoptosis [31]. There are different podocentric therapeutic options for DN and other glomerulopathies, such as angiotensinconverting enzyme (ACE) inhibitors or angiotensin receptor blockers (ARB), glucocorticoids, CNIs, and even Rituximab [32]. The apoptosis pathway is widely involved in many diseases, having effects on cell growth and differentiation. Some lines of evidence suggest that podocyte apoptosis is closely related to proteinuria in DN [33]. In the present study, we established a rat model of type 2 DN (T2DN) and studied the effects of FK506 on podocytes *in vivo* and *in vitro* following 8 weeks of treatment. We found higher 24 h uACR and abnormal structures of renal tissues in diabetic rats, including glomerular hypertrophy, foot process fusion, and thickened basement membrane. We also found abnormal TRPC6 activation and high glucose-induced podocyte apoptosis in the DM and HG groups, characterized by increased expression levels of cleaved-caspase-3 and bax/bcl-2 ratios, while FK506 reduced the extent of podocyte damage related to DN and attenuated the elevated expression levels of TRPC6 with improvement in the nephrin expression. Our findings are in line with those of previous studies that demonstrated the role of stimulation of high glucose and FK506 on podocytes [29, 34].

FK506, generally known as tacrolimus, is a fermentative product isolated from *Streptomyces*. It is a powerful immunosuppressive agent that is similar to macrolide antibiotics in terms of chemical structure. FK506 is a calcineurin inhibitor that interferes with calcium-dependent signaling pathways and that blocks the activation and proliferation of T cells by means of inhibiting production of cytokines such as interleukin-2 (IL-2). It is mostly used to prevent immunological rejection after organ transplantation. Recently, the relationship between FK506 and apoptosis has attracted increasing attention. A neurological study found that FK506 protected nerve cells from apoptosis while promoting axonal and nerve regeneration recovery in diffuse axonal injury (DAI) [35], possibly suggesting a new treatment for DAI. FK506 also exhibited antiapoptotic effects in many kidney diseases. In lupus nephritis (LN) and puromycin aminonucleoside- (PAN-) induced kidney damage, after administration of FK506, podocyte apoptosis was significantly decreased with reduced proteinuria [36, 37], alleviating the renal injury. Based on this, we proposed that FK506 would play an antiapoptotic role in DN. We found that FK506 significantly reversed the elevation of levels of cleaved-caspase-3 and bax/bcl-2 ratios in both DM rats and in podocytes exposed to high glucose levels, as well as promoting the nephrin expression. TUNEL assay and FCM results indicated that FK506 decreased apoptosis along with the TRPC6 expression both *in vivo* and *in vitro*. These results suggest that FK506 ameliorates the TRPC6 and apoptotic protein expression, thereby protecting podocytes from DN.

TRPC6 channels were associated with the development of diabetic complications such as DN. Recent studies further provided important evidence regarding the role of TRPC6 in the progression of DN; there are both positive and negative findings [38]. As a crucial component of podocytes, overactivation of TRPC6 drives dramatic influx of Ca^2+^ into podocytes, resulting in podocyte injury and apoptosis. CaN is a serine/threonine protein phosphatase regulated by the Ca^2+^/calmodulin; investigators have shown that regulation of the TRPC6 expression by angiotensin II requires TRPC6-mediated Ca^2+^ influx and the activation of CaN and nuclear factor of activated T cells (NFAT) [39]. In the present study, we found significantly lower expression of TRPC6, cleaved-caspase-3, and bax/bcl-2 ratios, along with lower apoptosis percentages after treating with the calcineurin inhibitor FK506.

Even though FK506 has antiapoptotic effects, further studies need to be carried out to explore the underlying mechanisms of FK506 against podocyte apoptosis. Lu et al. [40] found that high glucose elevated expression levels of TRPC6 and intracellular calcium in cultured podocytes but not in TRPC6 knockdown podocytes. TRPC6-dependent Ca^2+^ influx was shown to be involved in podocyte apoptosis induced by high glucose levels, and TRPC6 knockdown by siRNA interference blunted glucose-induced podocyte apoptosis [41]. Nijenhuis et al. showed that, in high glucose conditions, there was a deleterious positive feedback mechanism, in which angiotensin II induced the CaN/NFAT pathway that depends on Ca^2+^ influx through TRPC6 and, in turn, enhanced the expression of TRPC6 [39]. It has also been demonstrated that abnormal activation of calcineurin (CaN) contributes to promoting apoptosis in DN [42]. As a CaN inhibitor, FK506 may decrease apoptotic-associated protein expression and/or improve the expression of antiapoptotic molecules by inhibiting CaN and its positive feedback role on TRPC6.

In a DN animal model, FK506 restored podocyte-specific proteins, nephrin, and podocin, maintaining the integrity of GFB and as a result, leading to reduced proteinuria [21]. Abnormalities in immune cells, including T cells, B cells, and macrophages, partly account for the pathogenesis of DN, and FK506 is thought to relieve local infiltration of lymphocytes and macrophages in renal tissues as well as various inflammatory mediators because of its immunosuppressive effect.

Another important mechanism for DN is autophagy. This is a highly conserved lysosomal-dependent protein degradation process that is essential for various cells to maintain homeostasis [43]. For islet *β* cells, autophagy helps to preserve cell function under either normal or stress conditions [44]. Evidence has shown inhibition of autophagy in both rats and patients of T2DM [44–47]. Our previous studies suggested autophagy was significantly upregulated after FK506 intervention in T2DM rats, thereby protecting podocytes [24]. A network data analysis suggested that the pathogenesis of DN involves several molecules, including Toll-like receptors, cytokine receptor interactions, MAPK, TGF-*β*, and Ca^2+^ signaling pathways [48]. FK506 may exert its therapeutic effect via several targets, some of which overlap with the mechanisms discussed here.

In conclusion, the results of our experiment suggest that FK506 protects podocytes in DN by decreasing apoptosis, alleviating proteinuria, and delaying the progression of renal function deterioration; these effects may be mediated by decreased TRPC6 expression, perhaps providing new concepts for the use of FK506 as a DN therapy. Nevertheless, it should be noted that FK506 carries side effects such as elevated blood glucose levels, infection, and liver and kidney toxicity, depending on the dose and individual sensitivity. The amount of 0.5 mg/(kg·d) FK506 used for 8 weeks in the present study had no significant influence on hepatic function (ALT, AST) and WBC counting. Nevertheless, the safety of long-term treatment with FK506 in DN remains controversial, and further studies are needed to manage these problems.

## Figures and Tables

**Table 1:** General data of rats after 8 weeks of treatment (mean ± SEM).

Characteristics	NC (*n* = 10)	DM (*n* = 10)	FK (*n* = 10)
SBP (mmHg)	111.0 ± 4.3	138.3 ± 7.9^a^	130.9 ± 4.7^a^
BM (kg)	0.47 ± 0.07	0.62 ± 0.09^a^	0.60 ± 0.08^a^
KM/BM (g/kg)	3.08 ± 0.22	6.97 ± 0.31^a^	5.23 ± 0.17^ab^
FBG (mmol/L)	5.61 ± 0.61	19.61 ± 0.48^a^	18.90 ± 0.56^a^
FINS (mU/L)	18.76 ± 6.20	20.41 ± 5.17	19.26 ± 0.56
ISI	0.18 ± 0.02	0.05 ± 0.16^a^	0.03 ± 0.04^a^
CCr (mL/min)	1.48 ± 0.21	2.90 ± 0.23^a^	2.68 ± 0.31^a^
TG (mmol/L)	0.82 ± 0.23	4.13 ± 0.34^a^	3.82 ± 0.26^a^
TC (mmol/L)	2.46 ± 0.19	4.77 ± 0.66^a^	4.35 ± 0.44^a^
ALT (U/L)	46.3 ± 5.2	57.1 ± 1.4	58.7 ± 6.7
AST (U/L)	39.1 ± 6.5	41.3 ± 2.3	45.1 ± 5.3
WBC (×10^9^/L)	3.96 ± 0.63	4.72 ± 0.47	4.92 ± 0.74

^a^ *P* < 0.05 vs. NC group; ^b^*P* < 0.05 vs. DM group. SBP: systolic blood pressure; BM: body mass; KM/BM: kidney mass/body mass ratio; FBG: fasting blood glucose; FINS: fasting serum insulin; ISI: insulin sensitivity index; CCr: creatinine clearance; TG: triglyceride; TC: total cholesterol; ALT: alanine transaminase; AST: aspartate transaminase; WBC: white blood cell; NC: normal control group; DM: diabetes mellitus group; FK: FK506 group.

## Data Availability

The table and picture data used to support the findings of this study are included within the article.
